# Effects of platelet-rich plasma on human gingival fibroblast proliferation and migration *in vitro*

**DOI:** 10.1590/1678-7757-2018-0077

**Published:** 2018-06-25

**Authors:** Phuc Anh NGUYEN, Thuy Anh Vu PHAM

**Affiliations:** 1 University of Medicine and Pharmacy, Faculty of Odonto-Stomatology, Department of Periodontology, Ho Chi Minh City, Vietnam

**Keywords:** Platelet-rich plasma, Fibroblast, Cell proliferation, Migration

## Abstract

**Objective:**

This study evaluated the influence of platelet-rich plasma (PRP) on the behaviour of human gingival fibroblasts (hGFs), including fibroblast proliferation, migration and colony formation.

**Methods:**

PRP was obtained from the human peripheral blood of a healthy volunteer and then was diluted into platelet concentrations of 1%, 2% and 5%. The proliferation of hGFs was determined by two methods: (1) Cell-number counting with a haemocytometer method at days 1, 3, 5 and 7; (2) Colony-forming unit-fibroblast (CFU-F) assay at 2 weeks. The migration of hGFs was evaluated with scratch assay, then recorded digital images were analysed by Image-Analysis J 1.51j8 software to compare the remaining artificial wound areas between PRP groups at 0, 24 and 48 hours.

**Results:**

All hGFs that were cultivated in media with 1%, 2% and 5% PRP showed their ability to proliferate and migrate. Cell numbers incubated with 1% PRP increased significantly during the first three days and peaked at day 5, tending to be similar to their proliferation in complete medium. With concentrations of 2% and 5% PRP, hGFs outgrew and peaked at day 3, which was faster than with those in medium with 1% PRP. Especially, hGFs in the group 5% PRP proliferated with higher cell numbers than those in the other remaining groups at day 3. The hGF colony number that was formed in the group 5% PRP was significantly higher than those in the groups 1% and 2% PRP. Scratch assay showed hGFs in the groups 2% and 5% PRP almost filled the artificial wound and migrated more effectively than in the group 1% PRP at 24 hours, which was significant.

**Conclusion:**

In this study, perhaps the medium with 5% PRP is the dominant option, promoting the abilities of hGFs to heal wounds, because of its fast and effective impact on cell proliferation, colony formation and migration.

## Introduction

Recently, with the advancements in molecular biology, people have paid attention to the regeneration of periodontal cells by way of using biological intermediate substances for healing wounded tissue. Growth factors were used to stimulate cells in generating tissues in order to repair the body. As the quantity of growth factors at the wound increases, so does the number of cells activated to produce new tissue, helping wounds to heal more quickly and efficiently. Therein, polypeptide growth factors (PGFs) were thought to be one of the underlying elements in tissue engineering. They showed their important role in the growth and differentiation of cells involved in periodontal wound healing. Particularly, PGFs can regulate biological activities, including adhesion, migration, proliferation and differentiation of cells in bone and connective tissue^9^.

A convenient method that can help attract not only PGFs, but also epithelial growth factors, vascular endothelial growth factors, insulin-like growth factor, basic fibroblast growth factor and liver cell growth factor is using autogenous platelets. Platelet-rich plasma (PRP), the first generation of platelet gel used in periodontal regeneration therapy, was defined as using numbers of platelets 3–4 times higher than their standard in the body. Because of the high concentration of platelets in PRP, it leads to a higher number of growth factors available in wound tissue, which could activate cells to generate efficiently.^18^ ^,^ ^19^ Therefore, PRP therapy points in a very positive direction for periodontal regeneration with its fundamental role in promoting soft tissue healing.

In human gingiva, fibroblasts are the most common type of connective tissue cell. The estimation is that 1 cm^3^ gingival connective tissue has about 200 million fibroblasts, accounting for about 5% in volume. Fibroblasts are mesenchymal cells, which are responsible for producing most of the extracellular matrix tissue. This is very important in tissue repair and wound healing. Fibroblasts in different tissues have their own specific gene expression, growth and mobility characteristics. Due to their distinctive location and origin, gingival fibroblasts have their specific reactions to each wound, allowing them to cope optimally with the special conditions of oral and gingival lesions.^2^ ^,^ ^7^ ^,^ ^15^

Biologic features of *in vitro* human gingival fibroblasts (hGFs) depend on the PRP concentration in the medium; however, increasing the PRP concentration did not result in increasing cell numbers.^6^ ^,^ ^8^ ^,^ ^14^ Some studies showed that medium with a high concentration of PRP resulted in pH changes that negatively affected cell growth potential. Many studies in the last 5 years focused on the function of PRP at low concentration in comparison with those at high concentration.^6^ ^,^ ^22^ ^,^ ^24^ The studies of Tavassoli-Hojjati, et al.^22^ (2016) and Wang^24^ (2017) had similar results to each other, which had demonstrated that PRP low concentrations were more effective in hGFs features than the high ones. However, we didn’t find any studies that compared the effect between low concentrations of PRP and determined the best concentration for hGFs. The hypothesis of our study was that PRP low concentration (1%, 2%, 5%) has better affection on hGFs’ biologic features such as proliferation and migration. Thus, in order to explore potential applications of hGFs (particularly in periodontal healing), we might have conducted the first study about investigating the response of this cell type when cultured *in vitro* with low concentration PRP (1%, 2% and 5%) on these aspects: proliferation, colony formation and migration.

## Materials and methods

### Subculture of hGFs

Gingival tissue was collected from a healthy patient who underwent gingivectomy for aesthetic reasons. Culture and isolation of hGFs were conducted properly following the procedure of Physiology & Animal Biotechnology Department, University of Science in Ho Chi Minh City. These cells were subcultured to passage 4 in complete medium [Dulbecco’s modified eagle medium: nutrient mixture F-12 (DMEM/F12 - Sigma-Aldrich, MO, USA) supplemented with 10% foetal bovine serum (FBS - Sigma-Aldrich, MO, USA), 100 µg/mL streptomycin (Sigma-Aldrich, MO, USA) and 100 IU/mL penicillin (Sigma-Aldrich, MO, USA) at 37°C, 5% CO_2_ until 80% confluence]. We used hGFs at passage 4 in our study.

### PRP preparation

Human peripheral blood was collected from a healthy, non-smoking volunteer aged 20 to 30 years old, then was put into 3 test tubes (8.5 mL/tube) and centrifuged immediately at 2,000 rpm for 10 minutes. The upper yellow solution was transferred to an empty sterile tube and continuously centrifuged at 3,500 rpm for 5 minutes, and 10 mL of the upper yellow solution (platelet-poor plasma) was removed. PRP was activated by calcium chloride. Pellets were removed after 15 minutes, leaving behind activated PRP solution. PRP was diluted with DMEM/F12 to obtain different concentrations (1%, 2%, 5%) in experimental groups.

This research was approved by Ethical Committee of the University of Medicine and Pharmacy in Ho Chi Minh City with protocol number 225/DHYD-HD.

### Effect of PRP on hGF proliferation

To evaluate the role of PRP in cell proliferation, we conducted two methods: (1) Cell-number counting with a haemocytometer and (2) Colony-forming unit-fibroblast (CFU-F) assay. DMEM/F12 media with 1%, 2% and 5% PRP were the experimental groups. DMEM/F12 medium with and without 10% FBS were the positive and negative control groups, respectively, for comparison.

Haemocytometer cell counting method: hGFs were seeded in a 96-well plate (10^3^ cells/well) and cultured for 24 hours (h). Culture medium was replaced by PRP liquid extract and cultured for the next 1, 3, 5 and 7 days. In each indicated time point, cells were detached by trypsinisation (0.25% trypsin/ethylenediaminetetraacetic acid), stained with trypan blue and counted with a haemocytometer to determine the number of cells.

CFU-F assay: hGFs were seeded in a 6-well plate (2x10^3^ cells/well). Culture medium was replaced by PRP liquid extract and cultured on alternating days. At day 14, colonies in the Petri dishes were stained with a mixture of 6% glutaraldehyde and 0.5% crystal violet and observed.

### Effect of PRP on hGF migration

Scratch assay mimics cell migration during wound healing *in vitro* is suitable for studying the effect of PRP on cell migration. hGFs were seeded into 35 mm Petri dishes (5x10^4^ cells/dish) and cultured until 80% confluence. A scratch was produced in the monolayer on each dish using a pipette tip. Non-adherent cells were removed by washing once in PBS (phosphate-buffered saline). PRP extract was added in the dishes, and each was cultured for 24 h. At time points 0, 24 and 48 h, cell migration into the empty scratch surface under PRP extract conditions was monitored using phase-contrast microscopy and compared with cell migration under conditions of control media, then recorded by a digital camera. The area of wound healing was analysed using the Image-Analysis J 1.51j8 software (Wayne Rasband, National Institute of Mental Health, Bethesda, MD, USA).

### Data analysis

All experiments were repeated 3 times. For statistical analysis, independent samples comparison t-test, one-way ANOVA with Dunnett T3 (unequal variances) or Tukey HSD *post hoc* test (equal variances) were used for comparison between groups using SPSS v.23 (IBM, New York City, NY, USA) with the level of significance being 0.05.

## Results

### Effect of PRP on hGF proliferation

In general, in different cultured media of this experiment, hGFs were able to proliferate.

**Figure 1:**
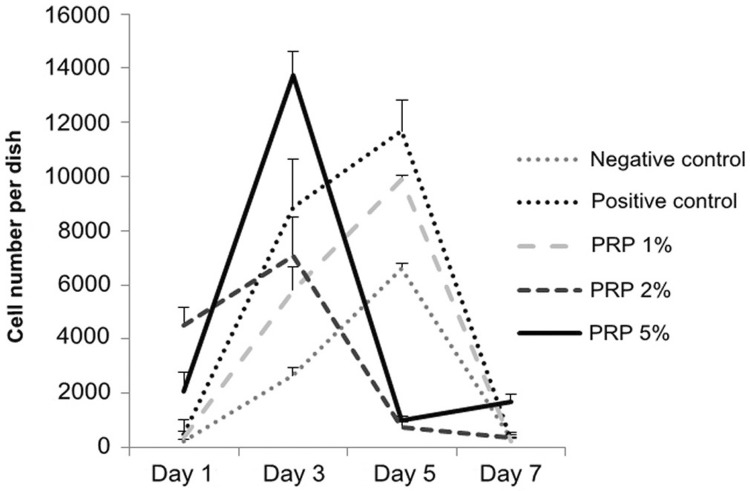
Cell number cultivated in experimental media at days 1, 3, 5 and 7 (Haemocytometer cell counting method)

The line of the group 1% PRP in Figure 1 was similar to those in the control groups, which showed hGF cell numbers increased from day 1 to day 3, then peaked at day 5. At day 3 and day 5, the quantity of cells in the group 1% PRP was significantly lower than in the positive control group and higher than in the negative one (p<0.05).

In the groups 2% and 5% PRP, hGFs had a different growth tendency compared with the group 1% PRP: cell number peaked at day 3, then decreased at day 5 (Figure 1).

**Figure 2:**
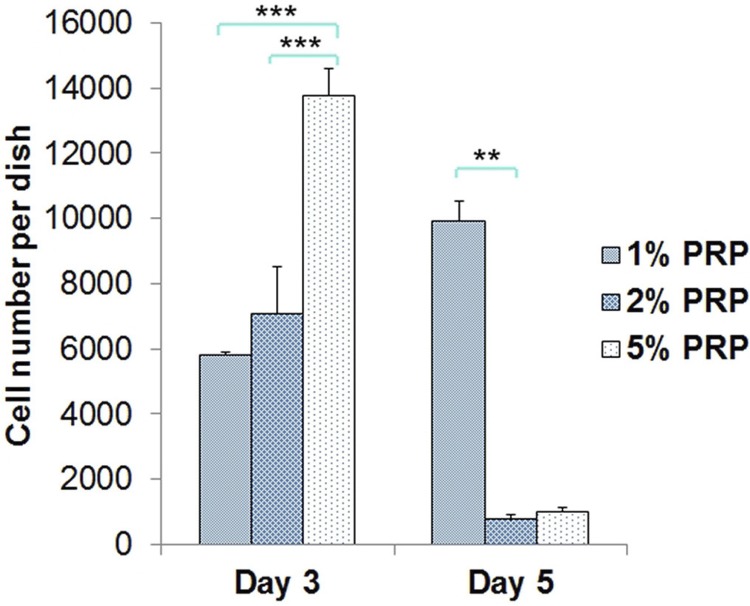
Cell number in media with PRP at day 3 and day 5 (** p<0.01, *** p<0.001)

At day 3, in the group 5% PRP, the quantity of hGFs was significantly higher than in the group 2% PRP (p<0.001) and 1% PRP (p<0.001). Cell number in the groups 1% and 2% PRP were not significantly different from each other (p=0.63). At day 5, cell number of the group 1% PRP continuously augmented and was significantly higher than of 2% PRP (p=0.003) and 5% PRP (p=0.004). There was no difference in cell number between the groups 2% and 5% PRP (p=0.64) (Figure 2).

**Figure 3:**
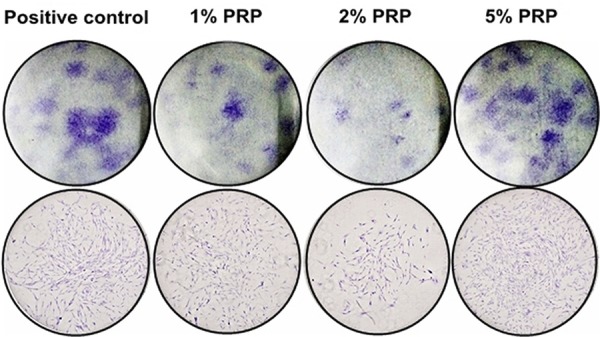
Clonogenic assay at day 14 (Upper line: observed by naked eye. Lower line: observed by microscope with 4x magnification)

**Figure 4:**
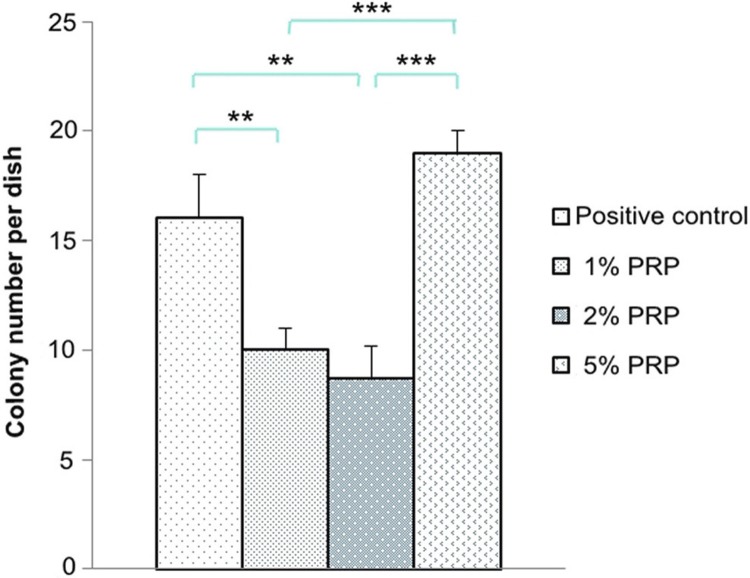
Colony number in experimental media at day 14 (** p<0.01, *** p<0.001)

After 14 cultivated days, colonies were formed in all the groups but the negative control (Figure 3). The colony number in the group 5% PRP was the highest with a significant difference in relation to those in the groups 1% and 2% PRP (p<0.001). There was no difference in colony number between the groups 1% and 2% PRP (p=0.68) (Figure 4).

### Effect of PRP on hGF migration

**Figure 5:**
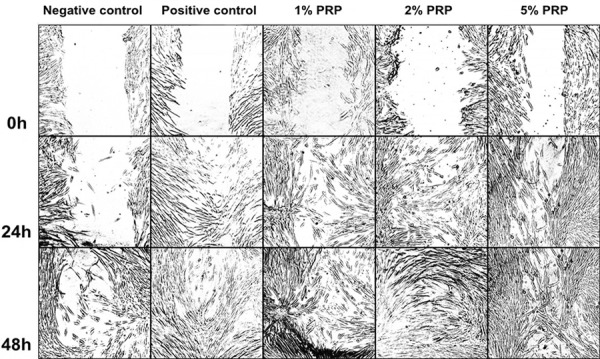
Scratch assay at 0, 24 and 48 hours (h) (Original magnification of these graphs was x10)5/9

Artificial wound closure in our experimental groups was performed in Figure 5 by taking snapshot pictures at different time points including 0 h, 24 h and 48 h. In all the experimental groups, the ability of hGFs to migrate into cell-free areas were shown after every 24 hours. At 24 h, morphology of partially migrating cells were clearly observed in all groups. And at 48 h, hGFs covered almost fully the surfaces at all, except for the negative control group whose cell-free area can be seen.

**Figure 6:**
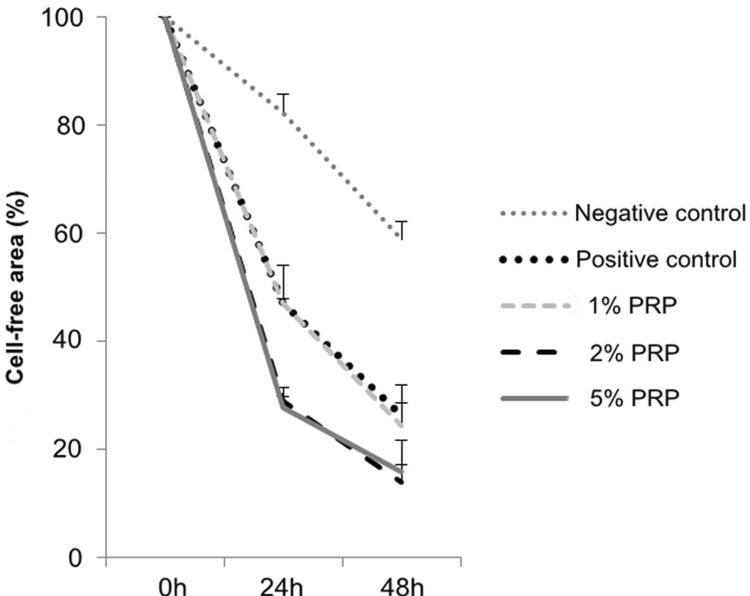
Cell-free area percentage in experimental media at 0, 24 and 48 hours (h)

In these media, for negative control, positive control, 1% PRP and 2% PRP the percentage of cell-free area decreased (with statistical significance) between the time points of 0 h and 24 h, 24 h and 48 h (p<0.001), which meant there was cell migration in these groups. Particularly, the area in the group 5% PRP significantly decreased in the period between 0 h and 24 h (p<0.01), but there was no difference between 24 h and 48 h (Figure 6).

The negative control group at both 24 h and 48 h had the largest cell-free area (p<0.05). There was no statistically significant difference between the group 1% PRP and the positive control group, or between the groups 2% and 5% PRP (p>0.05).

**Figure 7:**
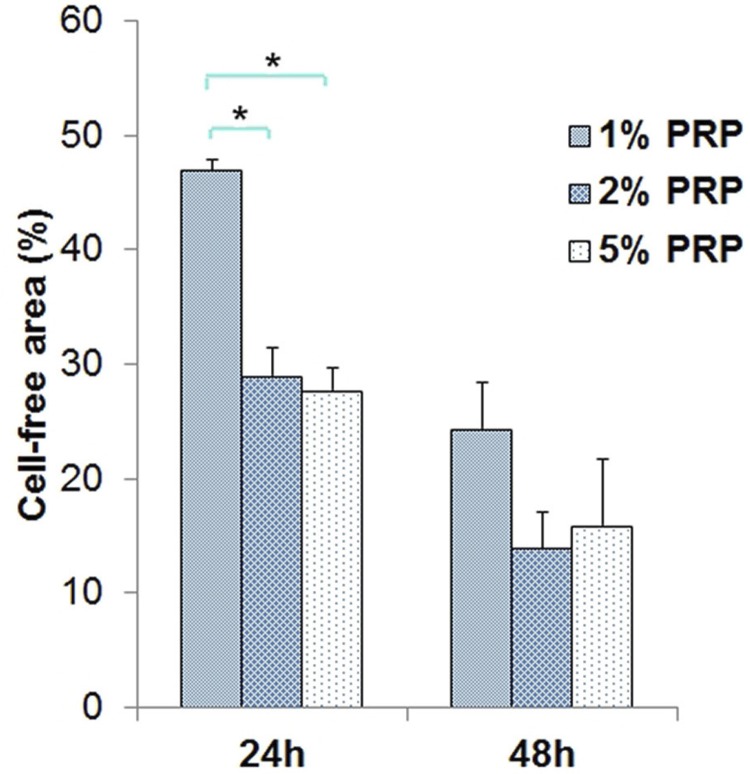
Cell-free area percentage in media with PRP at 24 hours and 48 hours (h) (* p<0.05)6/9

At 24 h, no significant difference was found between the groups 2% and 5% PRP (p=0.99). The cell-free area percentage in the groups 2% PRP (29.02%) and 5% PRP (27.73%) were significantly lower than in the group 1% PRP (p=0.001). At 48 h, although the cell-free area percentage in the group 1% PRP (24.37%) was higher than in the groups 2% PRP (13.93%) and 5% PRP (15.94%), this difference was not statistically significant (p>0.05) (Figure 7).

## Discussion

In recent years, the use of PRP became a popular therapy in order to provide growth factors for wounds and promote tissue regeneration. It had lots of advantages in comparison with other therapies, because PRP originated from autologous blood. The substantial advantages were its simplicity, safety and reasonable cost. Although the application of PRP was still being studied, some researchers showed evidence of a significant capacity for the increase of bone and soft tissue formation.^12^ ^,^ ^17^ However, some other researchers did not find any good points.^8^ ^,^ ^16^ Therefore, the effect of PRP on wound healing has been discussed until now. In this study, we used activated PRP, in which Ca^2+^ was added to release growth factors inside of platelets.

Heretofore, some recent studies have shown a PRP dose-dependent proliferation increase in the cells under investigation,^11^ ^,^ ^21^ with increasing PRP concentrations resulting in enhanced cell proliferation. Liu, et al.^14^ (2002) investigated the effect of different platelet concentrations on fibroblasts. They prepared maximally concentrated platelet preparations that were diluted in media to final concentrations of 8.8%, 17.5% and 35%. They found superior proliferation was obtained with the 8.8% and 17.5% preparations compared with the 35% concentration, because high PRP concentrations resulted in pH changes that negatively affected cell proliferation.^14^ Graziani^6^ (2006) found that although the effect of PRP on oral gingival fibroblasts and osteoblast proliferation was dose-dependent, increasing PRP concentrations did not result in increased cell proliferation. Han, et al.^8^ (2007) suggested the increase in cell proliferation induced by PRP containing 50–200 ng/mL TGF-β1 (12.5%–50% in v/v) was higher than in other PRP concentrations (with statistical significance), but there was no statistically significant difference within the range of these concentrations^8^.

Many studies in the last 5 years tended to focus on the function of PRP at low concentration. Their results almost showed PRP at low concentration had better effectiveness than at high concentration. For example, Tavassoli-Hojjati, et al.^22^ (2016) evaluated that media with 0.1% or 5% PRP were significantly more effective than those with 50% PRP.^22^ Wang, et al.^24^ (2017) found gingival fibroblasts had better proliferation in media with 1% and 5% PRP in comparison with those with 10% and 20% PRP. Some researchers thought that the quantity of receptors on the surface of cells was limited. Thus, when the amount of growth factors was larger than of receptors, they became exuberant and negatively affected cell function. The optimal platelet concentration was mainly dependent on the features of target cells.^6^ Therefore, in this study, we had chosen low PRP concentrations, including 1%, 2% and 5% to conduct experiments on proliferation, colony formation and migration of hGFs.

Cell proliferation assay was conducted over 7 days and 4 time points (day 1, day 3, day 5 and day 7) and recorded with the results showing the cell number threshold was reached at day 3 (for groups 2% and 5% PRP) or day 5 (for group 1% PRP). This is similar to Creeper, et al.^5^ (2012) conclusions, in which the various concentrations of PRP do not promote DNA synthesis in the short term (24 h), but over the longer term (5 days), they stimulate an increase in cell proliferation. At day 1, cells grew slowly, because they might not be adapted to their new environments; thus, the effect of PRP couldn’t reach the expected level. The period of 7 days was suitable for observing cell proliferation to its threshold and then contractionary phase due to the inhibition.

Our study showed that in 5% PRP-supplemented medium, hGFs had the best proliferation at day 3 when the cell number was significantly higher than in other media. This result was consistent with Tavassoli-Hojjati, et al.^22^ (2016), who investigated the concentrations of 0.1%, 5% and 50%. Their result pointed out that 5% PRP had the greatest effect on undifferentiated fibroblast proliferation, which was significant on the day 3. There was no significant difference between 0.1% PRP and the positive control during the first 3 days. The group with 50% PRP presented significantly lower cell proliferation compared with other experimental and control groups.^22^ Choi, et al.^3^ (2008) also showed similar results - a dilution of PRP to a level of 0.5%–5% would stimulate cell proliferation. Wang, et al.^24^ (2017) had a consistent point of view that the proliferation of cells peaked on day 7 of culture in the presence of 1% or 5% activated PRP and decreased in a dose-dependent manner in the presence of 10% or 20% activated PRP. The addition of 5% activated PRP to the culture medium maximally promoted cell proliferation.

Clonogenic assays were conducted to evaluate the effectiveness of specific agents on the survival and proliferation of cells. Tomar, et al.^23^ (2010) showed gingival mesenchymal stem cells, including hGF, had a more effective ability in self-renewal than bone marrow mesenchymal stem cells. Xu, et al.^20^ (2016) also showed gingival fibroblasts were considered as the source of seed cells in periodontal tissue engineering because of their advantages, such as easy isolation and expansion in experiments. Our study supported the effective clonogenic ability of hGF. Among the media with 1%, 2% and 5% PRP, the group 5% PRP seemed to be the best for hGFs to survive and self-renew.

In scratch assay, we have to control the proliferation of cells as an exclusion factor, therefore, cell migration results can be properly determined. The limitations of our study were that we did not use any growth inhibiting medications, such as aphidicolin, or reduce the serum concentration in cultivated media to restrict cell proliferation, as some methods to control cell number increase in previous studies.^10^ ^,^ ^13^ However, these methods were indicated differently for each type of cell. If we did not use the exact dose of medication or serum for particular cells in our studies, experiments could have failed because of cytotoxicity. We actually did not find any documentation about these doses for hGFs in the medical literature. In another way, the doubling time of hGFs in some previous studies was around 48 h.^25^ In our study, we examined cells migration at the time points 24 h and 48 h when the proliferation was considered not to be changed. Therefore, the results of migration in our study were proposed to be significant.

Cáceres, et al.^1^ (2008) told that to the best of their knowledge, this is the first study to show that growth factors can migrate and invade a reconstituted basement membrane in response to a chemotactic stimulus derived from PRP. Their results pointed out that concentrations of 100% (p<0.01), 50% (p<0.001) and 10% PRP (p<0.001) significantly enhanced migration compared with the negative control of 0% foetal calf serum. However, significant differences between the various concentrations of PRP^1^ were not found. Constanza, et al.^4^ (2012) in their study about gingival fibroblasts’ migration in nested matrices and cell invasion assay revealed that 25% PRP was likely to stimulate cell migration and invasion^4^. There were not many studies on the PRP concentration-dependence of hGF migration. Due to different experimental conditions, the results of other studies might not be similar to ours, particularly because the hGF migration of the groups 2% and 5% PRP was better than of the group 1% PRP and controls. However, basically the PRP potential for inducing hGFs to migrate and heal the wound is the consistent viewpoint between our research and the previous published ones. Recently, Xuzhu^24^ (2017) investigated the behaviour of gingival fibroblasts on titanium implant surfaces in combination with either injectable platelet-rich fibrin or PRP and found PRP induced a 250% significant increase relative to controls.^24^

## Conclusion

hGFs in media with PRP at concentrations of 1%, 2% and 5% demonstrated their abilities to proliferate and migrate. However, these abilities depended on the dose of PRP in media. In the proliferation experiment using the haemocytometer cell counting method, 5% PRP in medium supported hGFs to proliferate and peak in a short time; moreover, cell number in this group was significantly higher than in other groups at day 3. Also, 5% PRP was also the best concentration in the current study to stimulate hGFs to self-renew and form colonies. In scratch assay, 2% PRP and 5% PRP were the best factors to augment the migration of hGFs in comparison with 1% PRP and positive control groups. Eventually, this study suggested medium with 5% PRP was the dominant option, promoting the abilities of hGFs to heal wounds.
